# Molecular investigation of candidate genes for pyroptosis-induced inflammation in diabetic retinopathy

**DOI:** 10.3389/fendo.2022.918605

**Published:** 2022-07-25

**Authors:** Nan Wang, Lexi Ding, Die Liu, Quyan Zhang, Guoli Zheng, Xiaobo Xia, Siqi Xiong

**Affiliations:** ^1^ Eye Center of Xiangya Hospital, Central South University, Changsha, China; ^2^ Hunan Key Laboratory of Ophthalmology, Central South University, Changsha, China; ^3^ National Clinical Research Center for Geriatric Disorders, Xiangya Hospital, Central South University, Changsha, China

**Keywords:** pyroptosis, inflammatory death, diabetic retinopathy, competing endogenous RNA regulatory network, expression profiling by array

## Abstract

**Background:**

Diabetic retinopathy is a diabetic microvascular complication. Pyroptosis, as a way of inflammatory death, plays an important role in the occurrence and development of diabetic retinopathy, but its underlying mechanism has not been fully elucidated. The purpose of this study is to identify the potential pyroptosis-related genes in diabetic retinopathy by bioinformatics analysis and validation in a diabetic retinopathy model and predict the microRNAs (miRNAs) and long non-coding RNAs (lncRNAs) interacting with them. Subsequently, the competing endogenous RNA (ceRNA) regulatory network is structured to explore their potential molecular mechanism.

**Methods:**

We obtained mRNA expression profile dataset GSE60436 from the Gene Expression Omnibus (GEO) database and collected 51 pyroptosis-related genes from the PubMmed database. The differentially expressed pyroptosis-related genes were obtained by bioinformatics analysis with R software, and then eight key genes of interest were identified by correlation analysis, Gene Ontology (GO) enrichment analysis, Kyoto Encyclopedia of Genes and Genomes (KEGG) pathway analysis, and protein–protein interaction (PPI) network analysis. Then, the expression levels of these key pyroptosis-related genes were validated with quantitative real-time polymerase chain reaction (qRT-PCR) in human retinal endothelial cells with high glucose incubation, which was used as an *in vitro* model of diabetic retinopathy. Western blot was performed to measure the protein levels of gasdermin D (GSDMD), dasdermin E (GSDME) and cleaved caspase-3 in the cells. Moreover, the aforementioned genes were further confirmed with the validation set. Finally, the ceRNA regulatory network was structured, and the miRNAs and lncRNAs which interacted with CASP3, TLR4, and GBP2 were predicted.

**Results:**

A total of 13 differentially expressed pyroptosis-related genes were screened from six proliferative diabetic retinopathy patients and three RNA samples from human retinas, including one downregulated gene and 12 upregulated genes. A correlation analysis showed that there was a correlation among these genes. Then, KEGG pathway and GO enrichment analyses were performed to explore the functional roles of these genes. The results showed that the mRNA of these genes was mainly related to inflammasome complex, interleukin-1 beta production, and NOD-like receptor signaling pathway. In addition, eight hub genes—CASP3, TLR4, NLRP3, GBP2, CASP1, CASP4, PYCARD, and GBP1—were identified by PPI network analysis using Cytoscape software. High glucose increased the protein level of GSDMD and GSDME, as critical effectors of pyroptosis, in retinal vascular endothelial cells. Verified by qRT-PCR, the expression of all these eight hub genes in the *in vitro* model of diabetic retinopathy was consistent with the results of the bioinformatics analysis of mRNA chip. Among them, CASP4, GBP1, CASP3, TLR4, and GBP2 were further validated in the GSE179568 dataset. Finally, 20 miRNAs were predicted to target three key genes—CASP3, GBP2, and TLR4, and 22 lncRNAs were predicted to potentially bind to these 20 miRNAs. Then, we constructed a key ceRNA network that is expected to mediate cellular pyroptosis in diabetic retinopathy.

**Conclusion:**

Through the data analysis of the GEO database by R software and verification by qRT-PCR and validation set, we successfully identified potential pyroptosis-related genes involved in the occurrence of diabetic retinopathy. The key ceRNA regulatory network associated with these genes was structured. These findings might improve the understanding of molecular mechanisms underlying pyroptosis in diabetic retinopathy.

## Introduction

Diabetic retinopathy is an irreversible blindness disease characterized by retinal microvascular dysfunction (1). As one of the most prominent microvascular complications in advanced diabetes mellitus (DM), its incidence has been increasing worldwide and become a global public health problem (2, 3). The occurrence of diabetic retinopathy is closely related to the duration of disease and blood glucose level. Chronic exposure to hyperglycemia and other risk factors will lead to pathological retinal changes, such as breakdown of the retinal–blood barrier and aberrant retinal angiogenesis, which cause vision function impairment or even vision loss. It not only affects the quality of life in diabetic patients but also imposes a significant financial burden on the society (4). At present, the therapeutic options for diabetic retinopathy depend on the severity of the disease. It includes retinal laser photocoagulation, intravitreal injection of anti-VEGF drugs (5) or implantation of dexamethasone sustained-release agent (Ozurdex), and vitreoretinal surgery (6). However, some of the patients still suffer from a continuous decline of visual acuity after the aforementioned treatments due to progression of diabetic retinopathy. Therefore, elucidating the pathogenesis of diabetic retinopathy and providing treatment according to it might offer better therapeutic effects.

Pyroptosis is a new way of programmed cell death mediated by gasdermin (7). Differently from other ways of cell death, such as apoptosis and necrosis, pyroptosis is characterized by dependence on inflammatory caspase and the release of a large number of pro-inflammatory factors (8). According to the activation mechanism, pyroptosis can be divided into two signal pathways. The canonical pathway is characterized by the direct activation of caspase-1. It can cleave the N-terminal sequence of gasdermin D (GSDMD) which forms pores on the cellular membrane, followed by cytoplasmic swelling and membrane rupture. Meanwhile, activated caspase-1 can promote the release of inflammatory factors such as IL-18 and IL-1β to induce inflammatory response (9–11). In the non-classical pathway, inflammatory stimulators, such as lipopolysaccharide (LPS), can directly activate caspase-4/5/11 and other members of the caspase family. It can process GSDMD and indirectly activate caspase-1 to induce pyroptosis (12, 13). Under physiological conditions, pyroptosis is crucial for the maintenance of innate immunity and prevention of tumor occurrence (14), but accumulated dates have shown that abnormal pyroptosis is associated with cardiovascular diseases (15), nervous system disorders (16), and diabetic complications such as diabetic cardiomyopathy and diabetic retinopathy (17, 18). However, there is a little information concerning pyroptosis in diabetic retinopathy. Further exploration of the role of pyroptosis in diabetic retinopathy and uncovering the molecular mechanisms underlying it can provide a novel insight into the pathogenesis of diabetic retinopathy.

**Figure 1:**
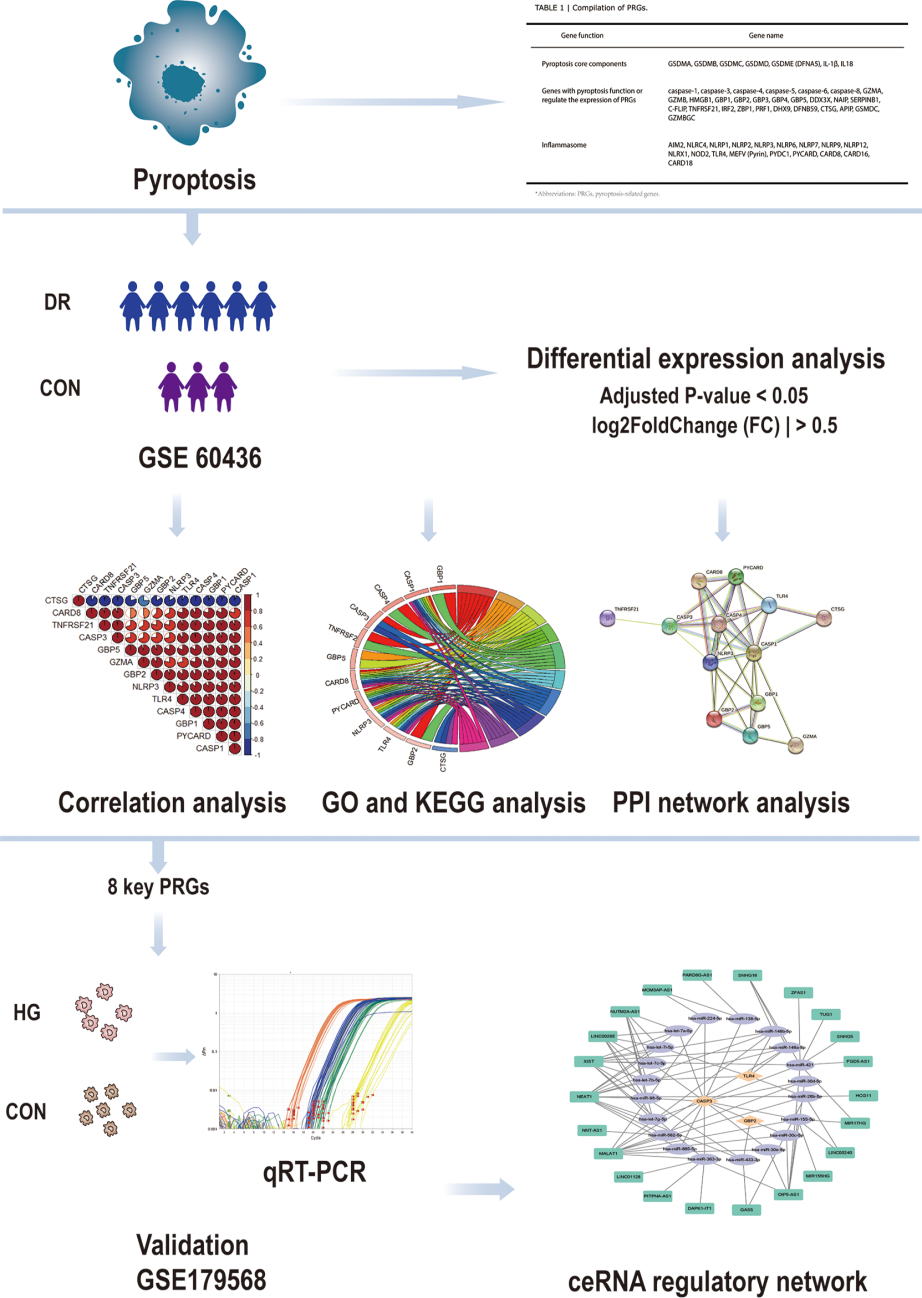
Design idea of this study. Downloaded GSE60436 dataset from the GEO database and 51 PRGs collected from the PubMed database. The R software was used to process the data, such as quality control, normalization, and background correction. A total of 13 differentially expressed PRGs in the dataset were identified by difference analysis, and GO and KEGG enrichment analyses were performed. At the same time, eight hub genes were identified by PPI analysis and then verified with qRT-PCR experiments and GSE179568. Finally, the specific mechanism of PRGs in DR was revealed by structuring a ceRNA regulatory network through multiMiR database and starbase database. GEO, Gene Expression Omnibus; PRGs, pyroptosis-related genes; PPI, protein–protein interaction; DR, diabetic retinopathy; qRT-PCR, quantitative real-time polymerase chain reaction; ceRNA, competing endogenous RNAs; GO, Gene Ontology; KEGG, Kyoto Encyclopedia of Genes and Genomes.

Ishikawa K et al. generated the dataset GSE60436 (19). They analyzed the gene profile expressed in the fibrovascular membranes (FVMs) associated with proliferative diabetic retinopathy by detecting the gene expression of the FVMs from patients with proliferative diabetic retinopathy. In this study, the GSE60436 dataset was used to explore potential pyroptosis-related genes associated with diabetic retinopathy. Through correlation analysis, Gene Ontology (GO) enrichment analysis, Kyoto Encyclopedia of Genes and Genomes (KEGG) pathway analysis, and protein–protein interaction (PPI) network analysis of differentially expressed genes (DEGs), we identified the pyroptosis-related genes that participate in diabetic retinopathy and verified them by the independent dataset GSE179568 and quantitative real-time polymerase chain reaction (qRT-PCR) in a diabetic model. Finally, we predicted the targeting microRNAs (miRNAs) and long non-coding RNAs (lncRNAs) that are correlated with the validated key pyroptosis-related genes and structured a competing endogenous RNA (ceRNA) regulatory network. It will lay the groundwork for revealing the mechanisms of pyroptosis in diabetic retinopathy and generate therapeutic insights (**Figure 1**).

## Materials and methods

### Microarray data and the exploration of PRGs

The GSE60436 microarray dataset deposited by Ishikawa K et al. was downloaded from the Gene Expression Omnibus (GEO) database (https://www.ncbi.nlm.nih.gov/geo/). The dataset contains three active FVMs and three inactive FVMs, which were obtained from proliferative diabetic retinopathy patients, and the RNAs from human retinas were purchased and were extracted from normal retinas pooled from 99 Caucasians. All samples were analyzed by GPL6884 platform (IlluminaHumanWG-6v3.0 expression beadchip), and R software (version 4.1.0) was used for data quality control, normalization, background correction, and subsequent analysis. The RNA-seq-based dataset GSE179568 was also obtained from GEO, including seven human retinal neovascularization (RNV) membranes, 10 macular pucker, and seven macular hole samples as controls. The search for pyroptosis-related genes was mainly carried out in the PubMed database (https://pubmed.ncbi.nlm.nih.gov/). Finally, 51 genes were collected (11, 20–26).

### Differential expression analysis and correlation analysis

We used “limma” package in R software to screen the differential genes in FVM samples of proliferative diabetic retinopathy patients and control groups. Genes with an adjusted *P*-value <0.05 and |log_2_ fold change| >0.5 were considered as differentially expressed genes, and one downregulated pyroptosis-related gene and 12 upregulated pyroptosis-related genes were identified. With *P*-value <0.05 and |log_2_ fold change| >1.0 as the threshold, 15 upregulated pyroptosis-related genes were obtained by analyzing the verification set GSE179568 with the same method. The correlation analysis of DEGs was carried out by using Spearman correlation in the “corrplot” package.

### Functional and pathway enrichment analysis

In order to evaluate the function of differentially expressed pyroptosis-related genes, we used GO enrichment analysis and KEGG pathway analysis in R software for functional and pathway enrichment analysis and visualization. The adjusted *P*-value <0.05 was taken as the standard with statistical significance.

### Construction of PPI network

The STRING database (https://string-db.org/) was used to construct the PPI network of DEG-encoded proteins (27), the threshold was set to a combined score ≥0.4, and the file in tsv format was downloaded. Then, Cytoscape software (version 3.9.0) was used to visualize the PPI network, and cytoHubba was used to excavate eight hub genes.

### Construction of ceRNA-regulating network

The “multiMiR” package in R software combines 14 databases, including the mirTarbase database (https://maayanlab.cloud/Harmonizome/resource/MiRTarBase) with experimental methods to verify the relationship, and uses this tool to predict the miRNAs of the pyroptosis-related genes of interest. All the lncRNA–miRNA interaction data were obtained in the starbase database (https://starbase.sysu.edu.cn/), and the target lncRNA was screened according to clipExpNum >7. Finally, visualization was carried out in Cytoscape software.

### Cell culture

Human retinal endothelial cells (HRECs) (CSC; Kirkland, WA, USA) were cultured in endothelial Complete Classic Medium (CSC; Kirkland, WA, USA) containing 10% fetal bovine serum (Gibco, USA). The culture medium was changed every 2 days. When the cells were 85–90% confluent, it was passaged at a ratio of 1:3. The *in vitro* diabetic model was induced by culturing HRECs with high-glucose medium containing 30 mM anhydrous glucose (Solarbio, Beijing, China) for 48 h, and the cells cultured in normal medium containing 5 mM glucose were used as control under the same conditions. In order to ensure the accuracy and stability of the results, 8–10 generations of cells were selected for follow-up study.

### RNA Extraction and qRT-PCR

According to the manufacturer’s plan, the total RNA of the experimental group and the control group was extracted by TRIzol reagent (Ambion, Carlsbad, CA). The concentration and the purity of RNA were measured by NanoDrop 2000c spectrophotometer (Thermo Fisher Scientific). Then, cDNA was reverse-transcribed from total RNA using UEIris II RT-PCR System (Suzhou Yuheng Biological Co., Ltd.) in strict accordance with the manufacturer’s instructions, and qRT-PCR was performed using 2× SYBR Green qPCR Master Mix (Suzhou Yuheng Biological Co., Ltd.). The sequence of the qRT-PCR primers is shown in **Supplementary Table S1**. Gene expression was detected by the 2^−ΔΔCt^ method. β-actin mRNA was selected as the internal control.

### Western blot analysis

HRECs were lysed with RIPA lysis buffer (Sangon Biotech, Shanghai, China) containing protease inhibitors and phosphatase inhibitors, and the protein concentrations were determined using the BCA kit (Thermo Fisher Scientific, Inc.). The samples were separated by 12% sodium dodecyl sulfate-polyacrylamide gel electrophoresis at 140 V and transferred to polyvinylidene membranes. Subsequently, the membranes were placed in 5% blocking buffer (bovine serum albumin) to block for 1 h, incubated with primary antibodies against CASP3 (Abcam, MA, USA), GSDMD (Abcam, MA, USA), GSDME (Abcam, MA, USA), and GAPDH (Abcam, MA, USA) at 4˚C overnight, followed by horseradish peroxidase-labeled secondary antibodies (Abcam, MA, USA) for 1 h at room temperature, and finally developed with an enhanced chemiluminescence kit for visualization. The collected images were examined using ImageJ software (version 6.0; Media Cybernetics, Inc.).

### Statistical analysis

Each *in vitro* experiment was repeated at least three times. Statistical analysis was performed using GraphPad Prism software (version 8.2.1). On the basis of the homogeneity of the variance test, Student’s *t*-test was performed, and *P <*0.05 was considered significant and statistically significant.

## Results

### Differential expression of pyroptosis-related genes in the FVMs of proliferative diabetic retinopathy patients and the retina of normal individuals

**Figure 2:**
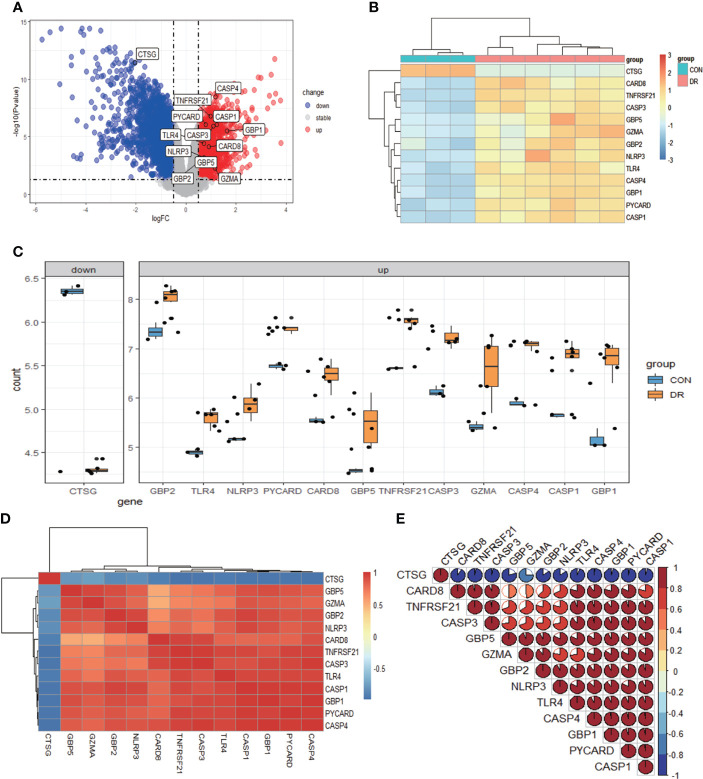
Differentially expressed PRGs in PDR and normal samples. **(A)** Volcano plot of 3,188 differentially expressed genes in the GSE60436 dataset. It contains 1,640 significantly upregulated genes, represented by red dots, and 1,548 significantly downregulated genes, represented by blue dots, whereas gray dots represent stably expressed genes. **(B)** Heatmap of 13 differentially expressed PRGs in the GSE60436 dataset. It contains one significantly downregulated gene and 12 significantly upregulated genes. The green bars represent control specimens from human retinas, denoted by “CON”, and the red bars represent specimens from PDR patients, denoted by “DR”. **(C)** Boxplot of 13 differentially expressed PRGs in FVM tissues of PRD patients and in RNA samples from human retinas. The blue bars represent control specimens from normal individuals, denoted by “CON”, and the yellow bars represent specimens from PDR patients, denoted by “DR”. Adjusted *P*-value <0.05 and |log_2_ fold change| >0.5. **(D, E)** Correlation analysis of 13 differentially expressed PRGs. There was a strong correlation among the 12 upregulated genes. PRGs, pyroptosis-related genes; PDR, proliferative diabetic retinopathy; FVM, fibrovascular membrane.

After quality control, normalization, and background correction of the data in the GSE60436 dataset, 3,188 genes with significant changes were obtained by using adjusted *P*-value <0.05 and |log_2_ fold change| >0.5 as thresholds, including 1,640 upregulated genes and 1,548 downregulated genes as shown in the volcano plot (**Figure 2A**). Due to the lack of a systematic database of pyroptosis-related genes, we collected 51 pyroptosis-related genes from PubMed database, including reported pyroptosis core components, genes with pyroptosis function or genes that regulate the expression of pyroptosis-related genes, and inflammasome that play an important role in cell pyroptosis, which were listed in **Table 1** (11, 20–26). One downregulated pyroptosis-related gene CTSG and 12 upregulated pyroptosis-related genes—CASP4, TNFRSF21, PYCARD, CASP1, CASP3, GBP1, TLR4, CARD8, NLRP3, GBP2, GBP5, and GZMA—were screened by intersecting these pyroptosis-related genes with genes in the GSE60436 database. These genes are annotated in the volcano plot (**Figure 2A**) and organized in **Table 2**. The expression of these genes was plotted as heatmap and box plot (**Figures 2B, C**). At the same time, we discussed the correlation of these genes, and the results showed that there was a correlation among the 13 pyroptosis-related genes (**Figures 2D, E**). Then, we further analyzed the differentially expressed genes between active FVMs and inactive FVMs according to the same thresholds and found an upregulated gene GZMB, indicating that GZMB may play an important regulatory role in different states of proliferative diabetic retinopathy.

**Table 1:** Compilation of pyroptosis-related genes.

Gene function	Gene symbol	Gene name
Pyroptosis core components	GSDMA	gasdermin A
	GSDMB	gasdermin B
	GSDMC	gasdermin C
	GSDMD	gasdermin D
	GSDME (DFNA5)	gasdermin E (DFNA5)
	IL-1β	interleukin 1 beta
	IL18	interleukin 18
Genes with pyroptosis function or regulate the expression of PRGs	CASP1	caspase-1
	CASP3	caspase-3
	CASP4	caspase-4
	CASP5	caspase-5
	CASP6	caspase-6
	CASP8	caspase-8
	GZMA	granzyme A
	GZMB	granzyme B
	HMGB1	high mobility group box 1
	GBP1	guanylate binding protein 1
	GBP2	guanylate binding protein 2
	GBP3	guanylate binding protein 3
	GBP4	guanylate binding protein 4
	GBP5	guanylate binding protein 5
	DDX3X	DEAD-box helicase 3 X-linked
	NAIP	NLR family apoptosis inhibitory protein
	SERPINB1	serpin family B member 1
	C-FLIP	cellular FADD-like interleukin-1β converting enzyme inhibitory protein TNF receptor superfamily member 21
	TNFRSF21	interferon regulatory factor 2
	IRF2	Z-DNA binding protein 1
	ZBP1	perforin 1
	PRF1	DExH-box helicase 9
	DHX9	guanylate binding protein 5
	DFNB59 (PJVK)	DFNB59 (pejvakin)
	CTSG	cathepsin G
	APIP	APAF1 interacting protein
Inflammasome	AIM2	absent in melanoma 2
	NLRC4	NLR family CARD domain containing 4
	NLRP1	NLR family pyrin domain containing 1
	NLRP2	NLR family pyrin domain containing 2
	NLRP3	NLR family pyrin domain containing 3
	NLRP6	NLR family pyrin domain containing 6
	NLRP7	NLR family pyrin domain containing 7
	NLRP9	NLR family pyrin domain containing 9
	NLRP12	NLR family pyrin domain containing 12
	NLRX1	NLR family member X1
	NOD2	nucleotide binding oligomerization domain containing 2
	TLR4	toll like receptor 4
	MEFV (Pyrin)	MEFV innate immuity regulator (Pyrin)
	PYDC1	pyrin domain containing 1
	PYCARD	PYD and CARD domain containing
	CARD8	caspase recruitment domain family member 8
	CARD16	caspase recruitment domain family member 16
	CARD18	caspase recruitment domain family member 18

51 pyroptosis-related genes were collected from the PubMed database (11, 20–26).

**Table 2:** The 13 differentially expressed pyroptosis-related genes in diabetic retinopathy samples compared to normal samples.

Gene symbol	logFC	Changes	P-value	Adjusted P-value	Gene id
CTSG	-2.04863800	Down	3.474745e-12	3.535901e-09	1511
CASP4	1.18248927	Up	3.190990e-09	5.237341e-07	837
TNFRSF21	0.97602261	Up	1.671004e-07	8.553391e-06	27242
PYCARD	0.77897161	Up	8.722862e-07	2.709519e-05	29108
CASP1	1.23521922	Up	8.987948e-07	2.748238e-05	834
CASP3	1.08640598	Up	1.156639e-06	3.351356e-05	836
GBP1	1.64253837	Up	3.128202e-06	6.727089e-05	2633
TLR4	0.71026963	Up	3.722308e-05	4.113837e-04	7099
CARD8	0.91154823	Up	7.318641e-05	6.827511e-04	22900
NLRP3	0.72061228	Up	6.323034e-04	3.673396e-03	114548
GBP2	0.67696609	Up	6.970046e-04	3.974848e-03	2634
GBP5	0.96463926	Up	2.668633e-03	1.181312e-02	115362
GZMA	1.16170618	Up	5.368367e-03	2.079660e-02	3001

PRGs, pyroptosis-related genes; DR, diabetic retinopathy; FC, fold change.

### Functional and pathway enrichment analysis of differentially expressed pyroptosis-related genes

**Figure 3:**
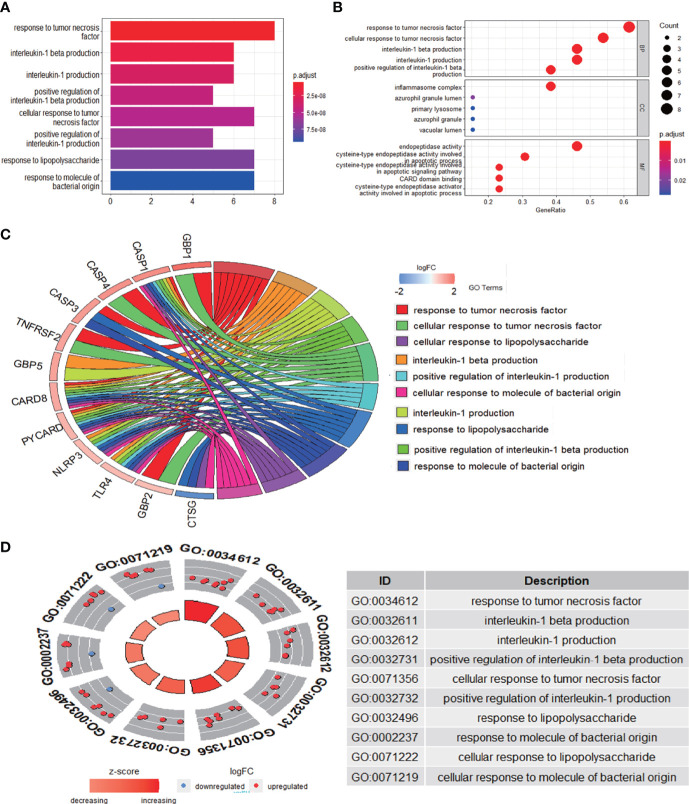
GO enrichment analysis of 13 differentially expressed PRGs. **(A)** Bar plot of enriched GO terms. **(B)** Bubble plot of enriched GO terms. **(C)** Chordal graph of enriched GO terms. **(D)** Eight diagrams of enriched GO terms. It contains three aspects—BPs, CCs, and MFs—and shows the specific genes involved in each GO term. GO, Gene Ontology; PRGs, pyroptosis-related genes; BPs, biological processes; CCs, cellular components; MFs, molecular functions.

**Figure 4:**
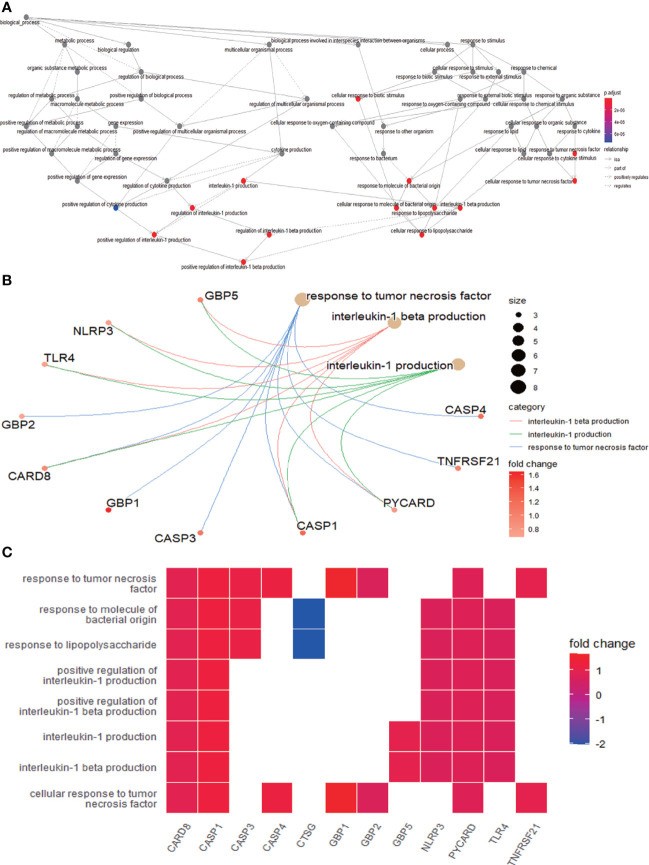
Further analysis of the results obtained by GO enrichment analysis. **(A)** Relationship between the pathways obtained by GO enrichment analysis. **(B)** Common genes among the three most prominent pathways. Each path is represented by lines of different colors. **(C)** Heatmap-like functional classification. The enrichment relationships of differentially expressed PRGs in the eight most significant pathways are shown. GO, Gene Ontology; PRGs, pyroptosis-related genes.

**Figure 5:**
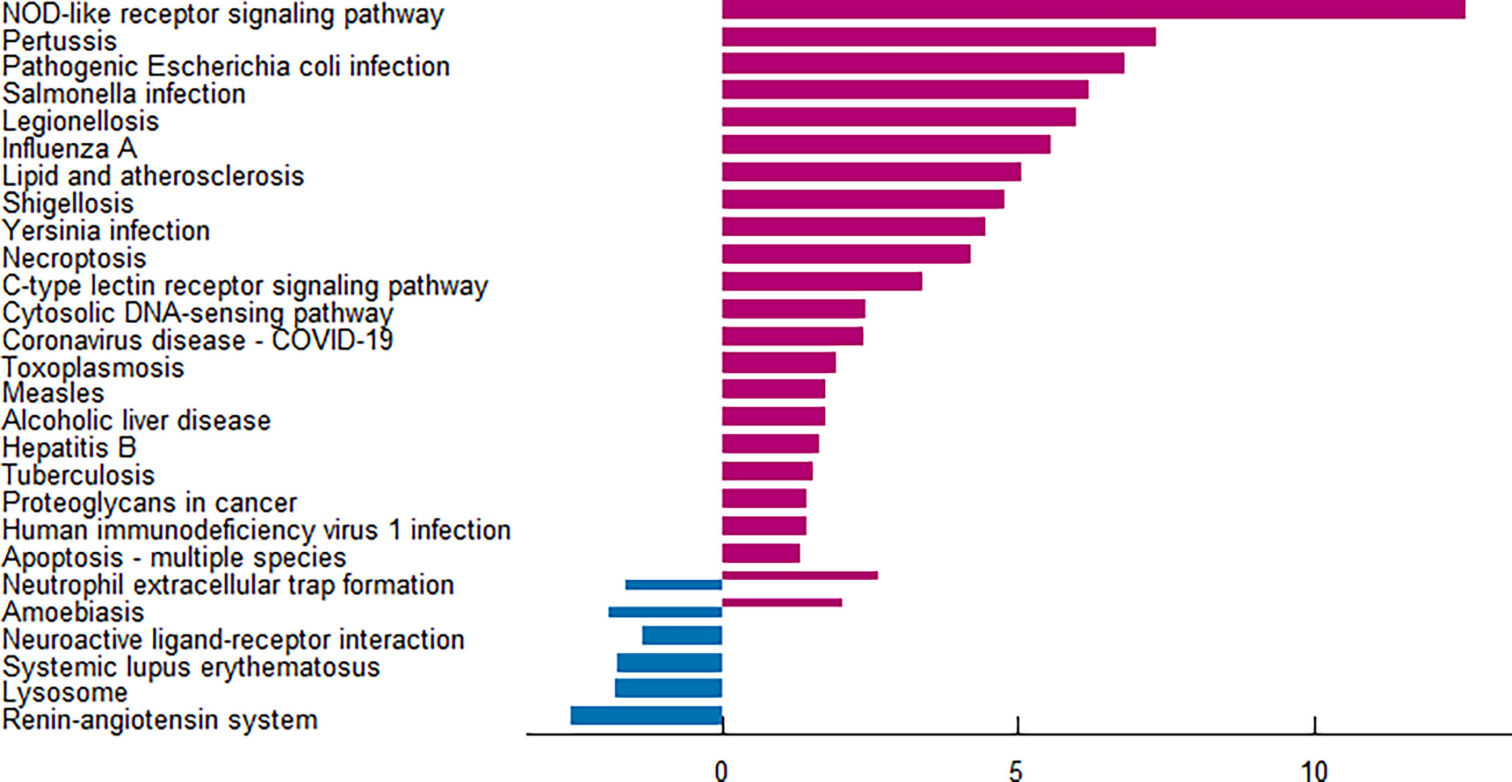
KEGG pathway analysis of 13 differentially expressed PRGs. According to the adjusted *P*-value, a total of 67 pathways were reported. KEGG, Kyoto Encyclopedia of Genes and Genomes; PRGs, pyroptosis-related genes.

The enrichment analysis was performed on the differentially expressed pyroptosis-related genes to further understand their potential molecular functions and signaling pathways. The GO analysis showed that 383 GO terms were significantly enriched, including 331 biological processes, five cellular components, and 47 molecular functions, involving interleukin-1 beta production, inflammasome complex, and CARD domain binding, indicating that these pyroptosis-related genes play a role in diabetic retinopathy through pyroptosis and inflammatory response (**Figures 3A–D**). Subsequently, we analyzed the relationship between these pathways, and the result is shown in **Figure 4A**. Then, we further analyzed the common genes of the first three pathways to clarify the relationship between the differentially expressed pyroptosis-related genes and these pathways (**Figure 4B**). The heatmap-like functional classification showed the specific enrichment of these genes in the first eight pathways (**Figure 4C**). At the same time, 67 significantly enriched pathways were obtained by KEGG analysis, indicating that these differentially expressed pyroptosis-related genes may play a key role in NOD-like receptor signaling pathway and other pathways (**Figure 5**). The most significant pathways obtained by GO analysis and KEGG analysis were shown in **Table 3**.

**Table 3:** Top 7 GO terms and top 3 KEGG pathway analyses for the differentially expressed PRGs.

Pathway id	Category	Description	Count	Genes	P-value
GO:0061702	CC	inflammasome complex	5	CASP4, PYCARD, CASP1, CARD8, NLRP3	3.36E-13
GO:0034612	BP	cysteine-type endopeptidase activity involved in apoptotic process	8	CASP4, TNFRSF21, PYCARD, CASP1, CASP3, GBP1, CARD8, GBP2	7.52E-12
GO:0032611	BP	interleukin-1 beta production	6	PYCARD, CASP1, TLR4, CARD8, NLRP3, GBP5	5.09E-11
GO:0032612	BP	interleukin-1 production	6	PYCARD, CASP1, TLR4, CARD8, NLRP3, GBP5	1.30E-10
GO:0097153	MF	cysteine-type endopeptidase activity involved in apoptotic process	4	CASP4, PYCARD, CASP1, CASP3	2.06E-10
GO:0032731	BP	positive regulation of interleukin-1 beta production	5	PYCARD, CASP1, TLR4, CARD8, NLRP3	2.43E-10
GO:0071356	BP	cellular response to tumor necrosis factor	7	CASP4, TNFRSF21, , CASP1, GBP1, CARD8, GBP2	3.46E-10
hsa04621	KEGG	NOD-like receptor signaling pathway	9	CASP4, PYCARD, CASP1, GBP1, TLR4, CARD8, NLRP3, GBP2, GBP5	8.37E-13
hsa05133	KEGG	Pertussis	5	PYCARD, CASP1, CASP3, TLR4, NLRP3	7.53E-08
hsa05130	KEGG	Pathogenic Escherichia coli infection	6	CASP4, PYCARD, CASP1, CASP3, TLR4, NLRP3	2.77E-07

*GO, Gene Ontology; KEGG, Kyoto Encyclopedia ofGenes and Genomes; PRGs, pyroptosis-related genes.

### Construction of PPI network

**Figure 6:**
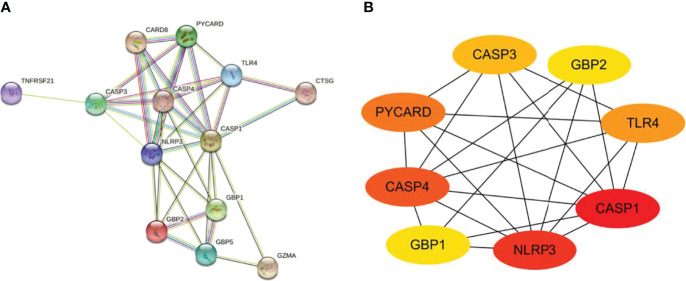
Construction of PPI network and identification of hub genes. **(A)** The PPI network of 13 differentially expressed PRGs was constructed by using String database. It contains 13 nodes and 35 edges. The average node degree is 5.38, and the PPI enrichment *P*-value is less than 1.0e-16. **(B)** First eight hub genes of the PPI network. First eight genes with the highest degree identified by Cytoscape software and CytoHubba. These genes are ranked in descending order from red to yellow. PPI, protein–protein interaction; PRGs, pyroptosis-related genes.

The PPI network is composed of individual proteins through the interaction between each other, which is of great significance for understanding protein function and interaction (27). In order to further explore the differentially expressed pyroptosis-related genes, we carried out PPI analysis. Firstly, the network files sorted out in R software are uploaded to String database, which is a network tool for exploring known and predicted protein–protein interactions (28). A PPI network with 13 nodes and 35 sides is obtained, and the average node degree is 5.38 (**Figure 6A**). Then, we used the cytoHubba in CytoScape software for further analysis, sequenced these differentially expressed pyroptosis-related genes by MCC mode, and screened the first eight hub genes, namely: CASP1, NLRP3, CASP4, PYCARD, TLR4, CASP3, GBP2, and GBP1 (**Figure 6B**, **Table 4**).

**Table 4:** PPI network of the top 8 hub genes.

Rank	Gene symbol	Score	Gene id	Gene name
1	CASP1	196	834	caspase-1
2	NLRP3	192	114548	NLR family pyrin domain containing 3 caspase-4
3	CASP4	168	837	PYD and CARD domain containing toll like receptor 4
4	PYCARD	144	29108	NLR family pyrin domain containing 3 caspase-4
5	TLR4	122	7099	PYD and CARD domain containing toll like receptor 4
6	CASP3	121	836	caspase-3
7	GBP2	48	2634	guanylate binding protein 2
8	GBP1	48	2633	guanylate binding protein 1

PPI, protein-protein interaction.

### Validation of pyroptosis-related genes

**Figure 7:**
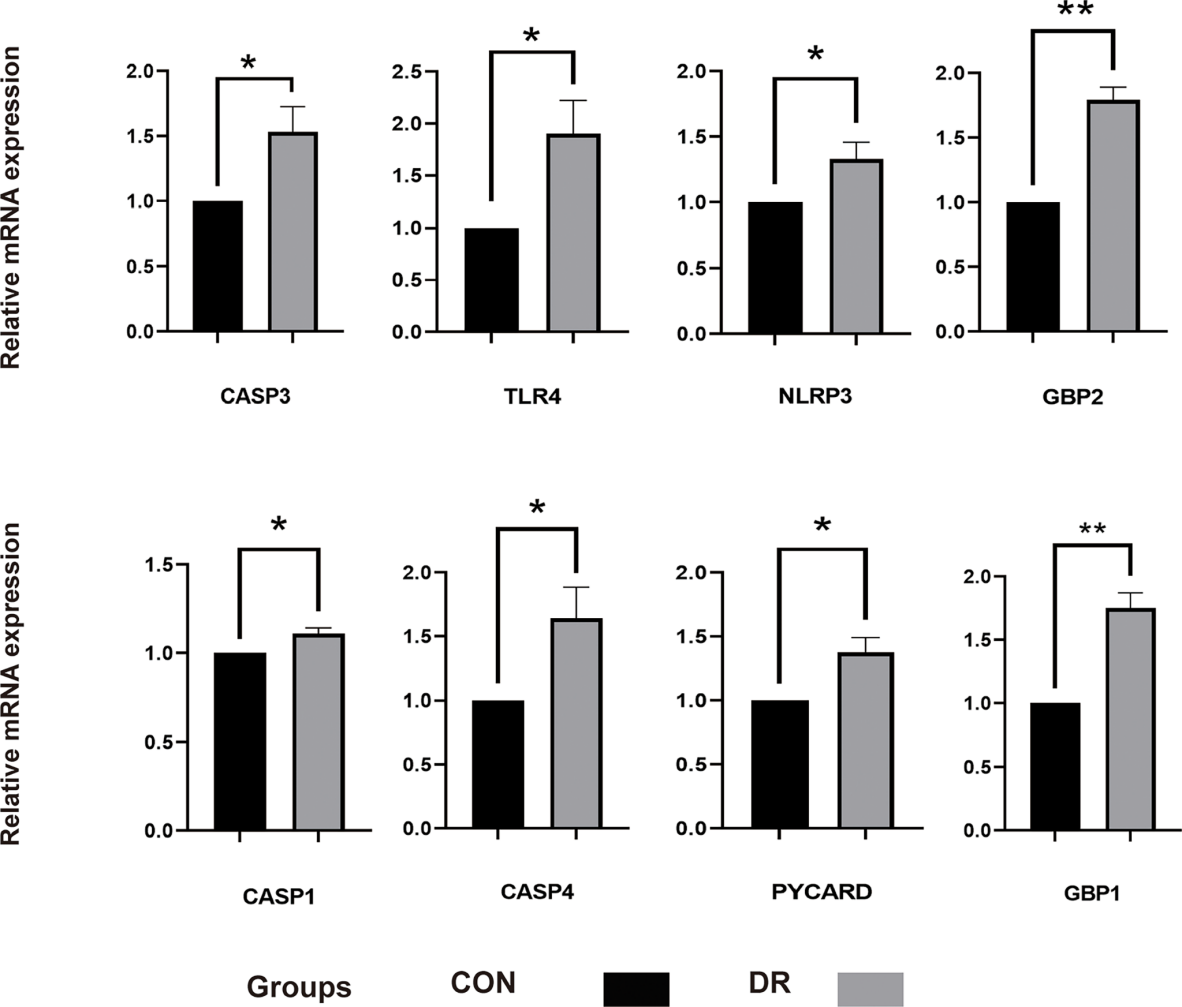
qRT-PCR experiment to verify the expression of PRGs of interest in the *in vitro* model. HRECs exposed to high glucose for 48 h were used as DR model, and HRECs cultured on normal culture medium were used as control. The *P*-values were calculated using a two-sided unpaired Student’s *t*-test. *P < 0.05; **P < 0.01. qRT-PCR, quantitative real-time polymerase chain reaction; PRGs, pyroptosis-related genes; HRECs, human retinal endothelial cells; DR, diabetic retinopathy.

**Figure 8:**
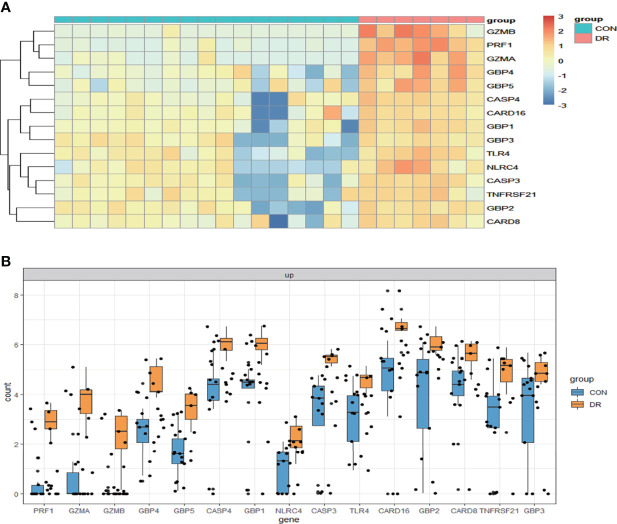
Validation of PRGs in the GSE179568 dataset. **(A)** Heatmap of 15 differentially expressed PRGs in the GSE60436 dataset. **(B)** Box plot of 15 differentially expressed PRGs in the RNV membranes of PRD patients and in the epiretinal membranes of macular pucker and macular hole samples. Adjusted *P*-value <0.05 and |log_2_ fold change| >1.0. PRGs, pyroptosis-related genes; RNV, retinal neovascularization; PDR, proliferative diabetic retinopathy.

**Figure 9:**
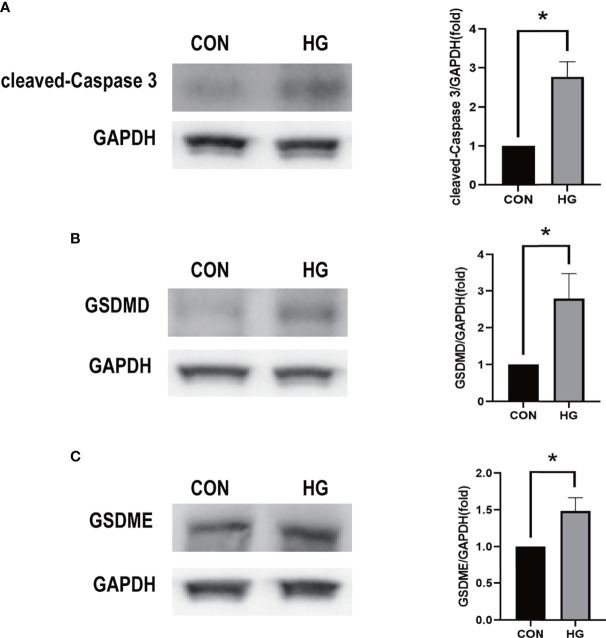
Western blot analysis of caspase-3 and gasdermin proteins in *in vitro* models. **(A)** Cleaved caspase-3 (17 kDa) was tested. GAPDH (36 kDa) was used as a control. **(B)** GSDMD (31 kDa) was examined. GAPDH was used as a control. **(C)** GSDME (55 kDa) was examined. GAPDH was used as a control. The experiments were repeated independently at least three times. *P < 0.05. CON, control group; HG, high-glucose group; GSDMD, gasdermin D; GSDME, gasdermin E.

To ensure the reliability of the results of the GSE60436 dataset analysis, we use qRT-PCR experiments and RNA-seq-based dataset GSE179568 for validation. Firstly, the expression levels of the eight key differentially expressed pyroptosis-related genes were further identified by qRT-PCR in the *in vitro* model of diabetic retinopathy. HRECs were cultured in high glucose medium (30 mmol/L anhydrous glucose) or normal-glucose medium. High-glucose-exposed HRECs were used as an *in vitro* model of diabetic retinopathy. Similar to the results of mRNA chip in the GSE60436 dataset, high glucose treatment significantly increased the mRNA expression levels of CASP3, TLR4, GBP2, CASP4, and GBP1 in HRECs. In addition, the expression levels of NLRP3, CASP1, and PYCARD were also increased (**Figure 7**). These results were in accordance with the bioinformatics analysis. Then, as described previously, we analyzed the 51 pyroptosis-related genes in the verification set with |log_2_ fold change| >1.0 as the threshold, and the trend of five pyroptosis-related genes was consistent with that of the hub genes analyzed by the GSE60436 dataset, including CASP4, GBP1, CASP3, TLR4, and GBP2 (**Figure 8**). In general, the results between the training set and the validation set are relatively consistent. Next, we detected the expression of caspase-3 and gasdermin proteins, the key executor of pyroptosis, in the *in vitro* model of diabetic retinopathy by western blotting. It showed that the protein levels of cleaved caspase-3, GSDMD, and gasdermin E (GSDME) were increased in human retinal endothelial cells after high glucose incubation, suggesting the occurrence of pyroptosis (**Figure 9**).

### Construction of ceRNA-regulating network

**Figure 10:**
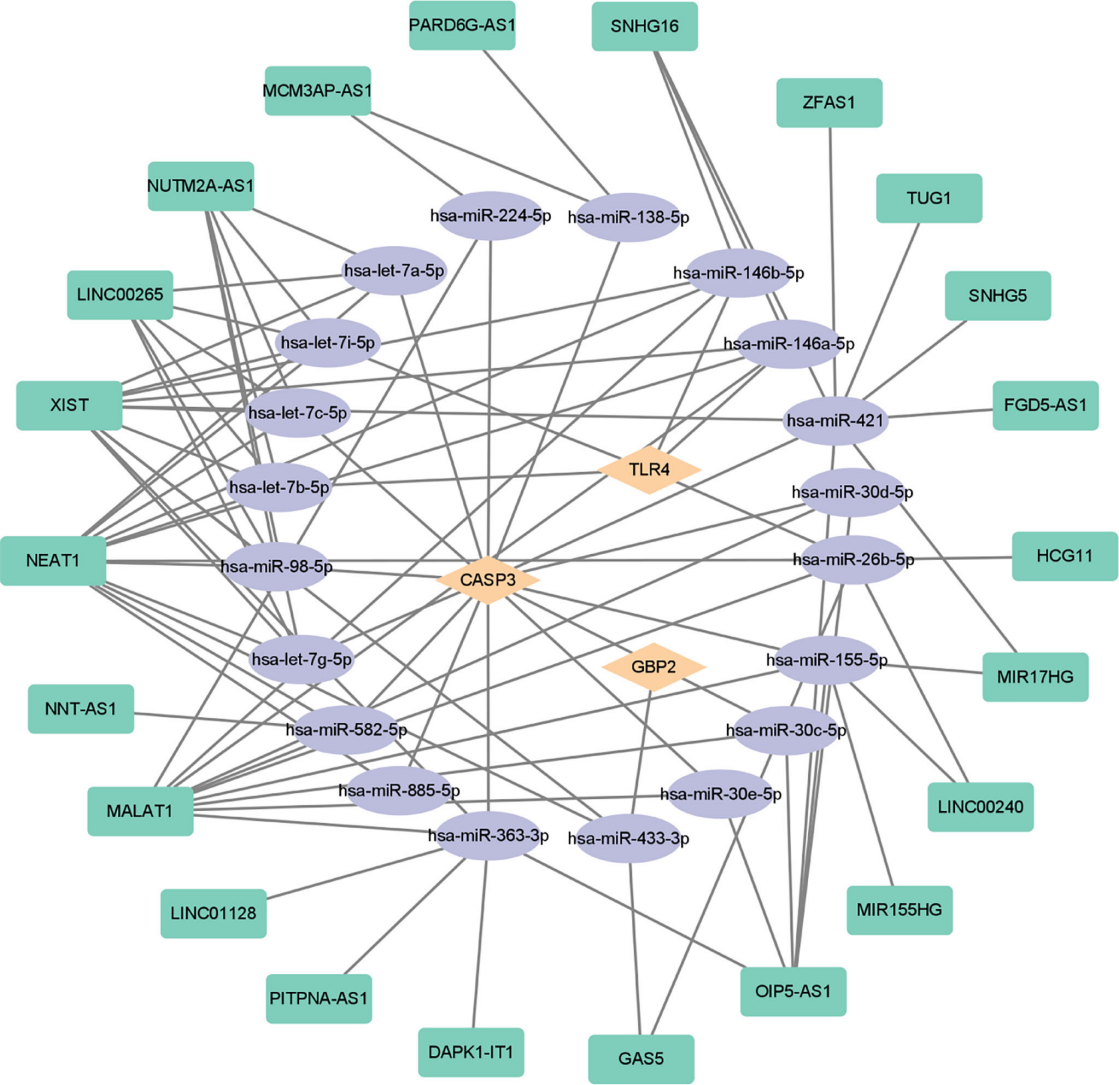
ceRNA-regulating networks. The yellow diamond represents the protein coding genes, the purple circle represents miRNAs, and the green rectangle represents lncRNAs. The black lines indicate the interaction of lncRNA–miRNA–mRNA. ceRNA, competing endogenous RNAs; miRNAs, microRNAs; lncRNAs, long non-coding RNAs.

In order to further explore the interaction between lncRNA, miRNA, and mRNA in diabetic retinopathy, we structured a ceRNA regulatory network. MultiMiR is a new miRNA–target interaction R package and database, which combines human and mouse records from 14 databases and includes mirTarbase databases with experimental methods to verify the interactions (29). Through this database, not only the miRNA that interacts with mRNA but also the targeted mRNA of miRNA can be predicted. We used the luciferase reporter assay as the screening criterion and used this tool to predict 20 miRNAs, including hsa-let-7a-5p, hsa-miR-433-3p, and so on (**Table 5**). Then, based on these miRNAs, we obtained 22 targeted lncRNAs through the starbase database. Finally, the three mRNAs, 20 miRNAs, and 22 lncRNAs were used to structure the ceRNA regulatory network, and the visualization was carried out in the CytoScape software (**Figure 10**).

**Table 5:** miRNAs and specic targeted mRNAs in ceRNA regulatory network.

mRNA	miRNA
CASP3	hsa-miR-421, hsa-let-7g-5p, hsa-miR-30e-5p, hsa-let-7c-5p, hsa-let-7a-5p, hsa-miR-363-3p, hsa-miR-30c-5p, hsa-miR-138-5p, hsa-miR-98-5p, hsa-miR-224-5p, hsa-miR-155-5p, hsa-miR-30d-5p, hsa-miR-582-5p, hsa-miR-885-5p
TLR4	hsa-let-7i-5p, hsa-miR-146b-5p, hsa-miR-26b-5p, hsa-let-7b-5p, hsa-miR-146a-5p
GBP2	hsa-miR-433-3p

miRNAs, microRNAs; ceRNA, competing endogenous RNAs.

## Discussion

It has been demonstrated that leukostasis, infiltration of neutrophil and macrophage, and activation of complements and microglial cells were found in the retinas of patients or animal model with diabetic retinopathy. Meanwhile, elevated levels of inflammatory cytokines and chemokines were detected in vitreous or aqueous humor from diabetic patients, indicating a critical role of inflammation in diabetic retinopathy (30, 31). Pyroptosis, known as cell inflammatory death, is accompanied by the release of a large number of pro-inflammatory factors, which is closely related to the pathogenesis of various chronic inflammatory diseases (8). Diabetic retinopathy is currently considered a chronic inflammatory disease. Thus, the involvement of pyroptosis-induced inflammation in diabetic retinopathy draws more and more attention. However, the molecular mechanisms underlying pyroptosis in diabetic retinopathy are unclear and need further investigations (32).

Bioinformatics methods have been widely used for the exploration of key pathogenic factors and potential therapeutic targets in diabetic retinopathy (33–35). To the best of our knowledge, bioinformatics analysis of pyroptosis-related genes in diabetic retinopathy has not been reported. In the current study, 13 differentially expressed pyroptosis-related genes in diabetic retinopathy were identified by bioinformatics for the first time, and eight of the most critical genes were identified by PPI analysis. At the same time, we performed GO analysis and KEGG analysis on these 13 differentially expressed pyroptosis-related genes to clarify the enrichment relationship between these genes and related pathways. The results showed that these genes were enriched in inflammasome complex, interleukin-1 beta production, and other pathways, confirming the role of pyroptosis in diabetic retinopathy.

Based on the results of the bioinformatics analysis, we identified eight most interesting pyroptosis-related genes according to PPI analysis and enrichment analysis. HRECs were incubated with high-glucose medium to mimic diabetic retinopathy *in vitro*. qRT-PCR was used for the verification of selected pyroptosis-related genes in a diabetic retinopathy model. The results showed that the change of mRNA level of CASP3, TLR4, NLRP3, GBP2, CASP1, CASP4, PYCARD, and GBP1 was consistent with that of the bioinformatics analysis of the mRNA chip. Among them, CASP3, TLR4, GBP2, CASP4, and GBP1 are verified in the validation set. We also showed that the cleaved CASP3 was upregulated in the *in vitro* model of diabetic retinopathy (**Figure 9A**). Several reports have shown that hyperglycemia can trigger the retinal inflammatory responses by abnormal metabolic pathways. It is accompanied by the activation of inflammasomes such as NLRP3 and TLR4, which play an important role in cell pyroptosis (36–38). Meanwhile, it has been reported that these potential pyroptosis-related genes mediated pyroptosis mainly due to the release of inflammatory cytokines such as IL-1β and IL-18 and lactate dehydrogenase, and the activation of GSDMD promotes pore formation in cells. Pyroptosis is associated with the damage of retinal vascular endothelial cells, retinal pericytes, and retinal pigment epithelium (RPE) cells in diabetic retinopathy (39–44). In the current study, both GSDMD and GSDME were increased in the high-glucose-treated retinal vascular endothelial cells, which is consistent with previous findings, indicating that hyperglycemia can trigger pyroptosis in retinal vascular endothelial cells. Destruction of retinal vascular endothelial cells by hyperglycemia leads to blood–retinal barrier damage and occlusion of the retinal capillary, which cause macular edema and pathological retinal neovascularization (45, 46). Rescuing the disrupted retinal vascular endothelial cells is the therapeutic strategy for diabetic retinopathy. Therefore, further elucidating the function of the aforementioned pyroptosis-related genes in retinal vascular endothelial cells will provide a deep insight into the mechanism underlying diabetic retinopathy.

Recently, increasing data demonstrated that lncRNA- and miRNA-mediated pyroptosis is involved in the occurrence of diabetic retinopathy. miRNA miR-590-3p, which has been proved to play an active role in inflammation (47), could directly target NLRP1 and NOX4 and inhibit pyroptosis in diabetic retinopathy through the NOX4/ROS/TXNIP/NLRP3 pathway (42). METTL3, as the key methyltransferase for m^6^A RNA methylation, reduced the pyroptosis level in a diabetic model by targeting the miR-25-3p/PTEN/Akt signal cascade with the assistance of DGCR8 (43). CircZNF532 could also regulate pyroptosis in RPE cells from diabetic retinopathy patients by targeting the miR-20b-5p/STAT3 axis (44). Another study found that lncRNA MIAT was significantly upregulated in human retinal pericytes and promoted human retinal pericyte pyroptosis by regulating miR-342-3p targeting CASP1 (41). Therefore, we constructed the ceRNA-regulating network to figure out the reciprocal interaction between lncRNA and miRNA and its function in regulating pyroptosis-related gene expression. By using multiMiR database, we predicted 20 miRNAs to be involved in the modulation of three essential pyroptosis-related genes, such as CASP3, TLR4, and GBP2. Subsequently, we found a possible interaction between 22 lncRNAs and these 20 miRNAs. Based on these findings, we created the ceRNA-regulating network through the aforementioned 22 lncRNAs, 20 miRNAs, and three target mRNAs. Among these genes, TLR4 as a pattern recognition receptor is very important for the activation of NLRP3 inflammatory bodies (48). After activation, inflammatory bodies can bind to pro-caspase-1 through the binding protein ASC, activate caspase-1, and induce cell pyroptosis through the classical pathway. At the same time, in the non-classical pathway, although caspase-4/5/11 can be directly activated by LPS without the participation of receptors, it still needs to be completed with the assistance of caspase-1 activated by NLRP3. Both classical and non-classical pathways will induce pyroptosis by cleaving GSDMD (49, 50). GBP2 plays a dual role in the occurrence of pyroptosis. It not only promotes the activation of AIM2 inflammatory cytokines but also controls the recruitment of caspase-4 (51, 52). In recent years, accumulated evidence have shown that caspase-3, which is usually regarded as a marker of apoptosis, can play an important role in pyroptosis by cleaving GSDME, which is another new pathway of pyroptosis that is different from the classical and non-classical pathways (8, 53, 54). In this study, we confirmed that high glucose could upregulate the expression of caspase-3 and GSDME in the diabetic model, suggesting that CASP3-mediated pyroptosis is involved in the development of diabetic retinopathy, which has not been explored. Further studies are required to uncover it.

## Conclusions

In this study, the key pyroptosis-related genes in diabetic retinopathy, such as CASP3, GBP2, and TLR4, were preliminarily identified by bioinformatics analysis and confirmed by GSE179568 and qRT-PCR in a diabetic model. lncRNAs and miRNAs that are associated with these genes were further predicted (**Figure 10**). It provides a novel insight into the molecular mechanism of pyroptosis in the pathogenesis of diabetic retinopathy.

## Data availability statement

The dataset presented in this study can be found in online repositories. The names of the repository/repositories and accession number(s) can be found in the article/**Supplementary Material**.

## Author contributions

NW and LD analyzed the data and drafted the manuscript. DL, XX, QZ, and GZ edited and provided comments to improve the manuscript. SX designed this experiment and reviewed and revised the manuscripts. All the authors contributed to the article and approved the final manuscript.

## Funding

This study was financially supported by the National Natural Science Foundation of China (no. 81974137 to SX), the National Natural Science Foundation of China (no. 82070966 to LD), the Natural Science Foundation of Hunan Province (no. 2019JJ40507 to SX), and the Science and Technology Innovation Program of Hunan Province (no. 2021RC3026 to LD).

## Conflict of interest

The authors declare that the research was conducted in the absence of any commercial or financial relationships that could be construed as a potential conflict of interest.

## Publisher’s note

All claims expressed in this article are solely those of the authors and do not necessarily represent those of their affiliated organizations, or those of the publisher, the editors and the reviewers. Any product that may be evaluated in this article, or claim that may be made by its manufacturer, is not guaranteed or endorsed by the publisher.
