# Supplementary material for: Molecular investigation of candidate genes for pyroptosis-induced inflammation in diabetic retinopathy
Source: Front Endocrinol (Lausanne). 2022 Jul 25;13:918605. doi: 10.3389/fendo.2022.918605 (PMC9357938; doi:10.3389/fendo.2022.918605)
Supplement: Supplementary file 1 [file DataSheet_1.docx]

GSDMD：

**Marker**

**Con2**

**Con1**

**HG2**

**Con3**

**HG3**

**HG1**


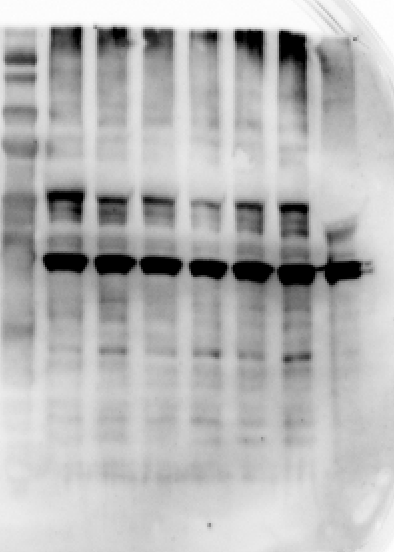


**GSDMD 31kD**

**Marker**

**Con3**

**HG1**

**Con2**

**Con1**

**HG3**

**HG2**


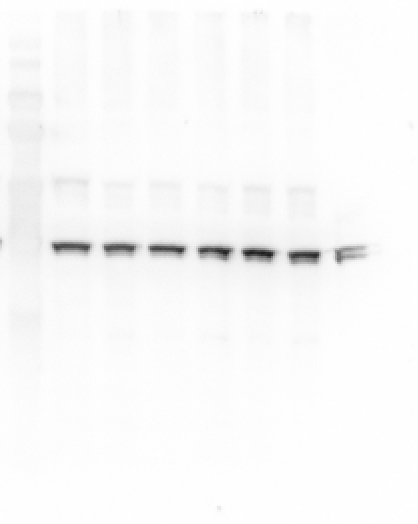


**GAPDH 36kD**

Con: control group, human retinal endothelial cells (HRECs) cultured on normal culture medium.

HG: High glucose group, HRECs exposed to high glucose for 24 hours were used as diabetic retinopathy model.

GSDMD (31 kDa) was examined. GAPDH was used as a control.

The experiments were repeated independently at least 3 times.

GSDME：

**Con3**

**HG2**

**Con2**

**HG1**

**Con1**

**HG3**

**Marker**


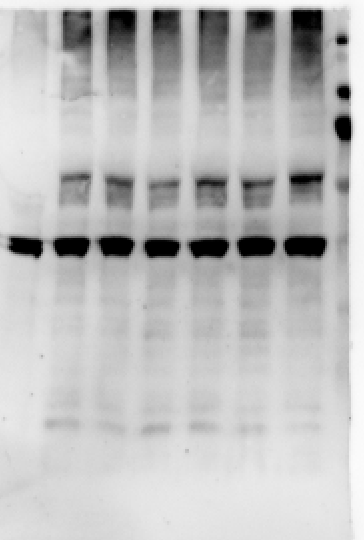


**GSDME 55kD**

**HG3**

**Con3**

**HG2**

**HG1**

**Con2**

**Con1**

**Marker**


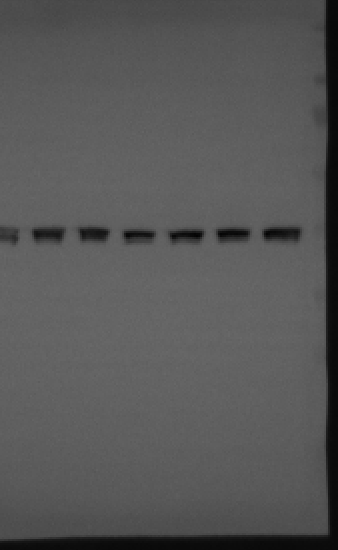


**GAPDH 36kD**

Con: control group, human retinal endothelial cells (HRECs) cultured on normal culture medium.

HG: High glucose group, HRECs exposed to high glucose for 24 hours were used as diabetic retinopathy model.

GSDME (55 kDa) was examined. GAPDH was used as a control.

The experiments were repeated independently at least 3 times.

cleaved-Caspase 3：

**HG5**

**HG4**

**HG3**

**HG2**

**HG1**

**Con5**

**Con4**

**Con3**

**Con2**

**Con1**

**Marker**


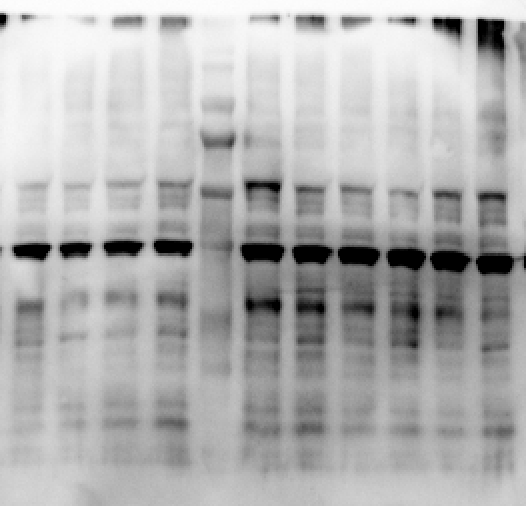


cleaved-Caspa**se**3 17kD **36kD**

**HG5**

**HG4**

**HG3**

**HG2**

**HG1**

**Con5**

**Con4**

**Con3**

**Con2**

**Con1**

**Marker**


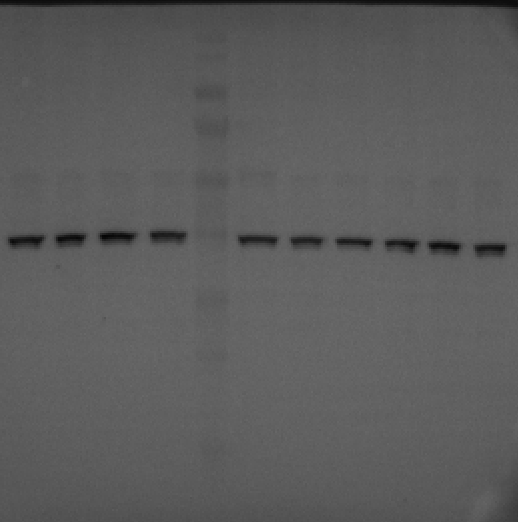


GAPDH 36kD

Con: control group, human retinal endothelial cells (HRECs) cultured on normal culture medium.

HG: High glucose group, HRECs exposed to high glucose for 24 hours were used as diabetic retinopathy model.

Cleaved-caspase3 (17 kDa) was tested. GAPDH (36 kDa) was used as a control.

The experiments were repeated independently at least 3 times.
